# Continuous Radio Amplification by Stimulated Emission of Radiation using Parahydrogen Induced Polarization (PHIP‐RASER) at 14 Tesla

**DOI:** 10.1002/cphc.201901056

**Published:** 2020-02-11

**Authors:** Andrey N. Pravdivtsev, Frank D. Sönnichsen, Jan‐Bernd Hövener

**Affiliations:** ^1^ Section Biomedical Imaging Molecular Imaging North Competence Center (MOIN CC) Department of Radiology and Neuroradiology University Medical Center Kiel Kiel University Am Botanischen Garten 14 24114 Kiel Germany; ^2^ Otto Diels Institute for Organic Chemistry Kiel University Otto Hahn Platz 5 24098 Kiel Germany

**Keywords:** coherent emission, NMR spectroscopy, para-hydrogen induced polarization, magnetic properties, RASER

## Abstract

Nuclear Magnetic Resonance (NMR) is an intriguing quantum‐mechanical effect that is used for routine medical diagnostics and chemical analysis alike. Numerous advancements have contributed to the success of the technique, including hyperpolarized contrast agents that enable real‐time imaging of metabolism in vivo. Herein, we report the finding of an NMR radio amplification by stimulated emission of radiation (RASER), which continuously emits ^1^H NMR signal for more than 10 min. Using parahydrogen induced hyperpolarization (PHIP) with 50 % para‐hydrogen, we demonstrated the effect at 600 MHz but expect that it is functional across a wide range of frequencies, e.g. 10^1^–10^3^ MHz. PHIP‐RASER occurs spontaneously or can be triggered with a standard NMR excitation. Full chemical shift resolution was maintained, and a linewidth of 0.6 ppb was achieved. The effect was reproduced by simulations using a weakly coupled, two spin‐1/2 system. All devices used were standard issue, such that the effect can be reproduced by any NMR lab worldwide with access to liquid nitrogen for producing parahydrogen.

## Graphical abstract

**PHIP‐RASER**: Continuous and coherent emission of NMR signal with a line width <1 ppb was observed for more than 10 minutes at 600 MHz with and without initial exciation using standard NMR equipment and parahydrogen induced polarization (PHIP‐RASER). PHIP‐RASER is based on radiation damping, the interaction between a strongly polarized sample and a high quality factor coil, and is expected to work at magnetic fields above 1 Tesla.

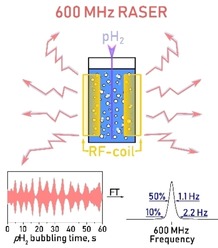

## 1 Introduction

The quest for a continuously emitting, high‐frequency, liquid‐state radio amplification by stimulated emission (RASER) at MHz frequencies and above is ongoing for more than a decade. Pioneering work demonstrated RASERs at low fields with resonance frequencies from 10 Hz to 50 kHz, based on ^3^He or ^129^Xe gases polarized with Spin Exchange Optical Pumping (SEOP),^1^, ^2^ for hours^3^, ^4^ or days. These techniques were used, among other things, for probing fundamental symmetries.^5^

A 400 MHz RASER was enabled by Dissolution Dynamic Nuclear Polarization (dDNP),^6^, ^7^ although only for a single shot experiment: RASER signal bursts were observed for three seconds after a hyperpolarized sample was poured into a cavity.^8^ Continuous emission of the NMR signal is not feasible with this approach because of relaxation and long sample preparation time.

Closest to a continuous emission at high frequency was likely a ^129^Xe RASER, where 11, 139 MHz bursts were observed at 11.7 T for 8.5 min. Because the polarization was not refreshed, the amplitude was continuously decaying. Interestingly, the magnetization of the sample was so strong that its Larmor frequency was elevated by 3 Hz at the beginning of the experiment due to strong distant dipolar fields of the sample itself.^9^

In a recent breakthrough, Süfke et al.^10^ demonstrated a continuous NMR RASER based on a combination of innovative hardware^11^ the continuous hyperpolarization^37^, ^38^ provided by Signal Amplification By Reversible exchange^12^ (SABRE): SABRE‐RASER.^13^ Using a sophisticated setup and continuously renewing polarization, a spectral resolution of 0.6 Hz was achieved at ≈3.8 mT (≈4 ppm). This unprecedented resolution of J‐couplings was reported, but chemical shift resolution was not possible because of the low magnetic field.^10^, ^13^ A transfer of this method to higher fields was not feasible because the level anticrossing (LAC) is required for spontaneous polarization transfer to occur (for ^1^H, ≈mT).^14^, ^15^ Spontaneous SABRE was reported at higher fields, too, but only for very small polarization.^16^, ^17^ Continuous high‐field Radio Frequency (RF) driven SABRE polarization transfer techniques still provide moderate and semi‐continuous polarization.^18^, ^19^, ^20^

## 2 Results and Discussion

**Figure 1:**
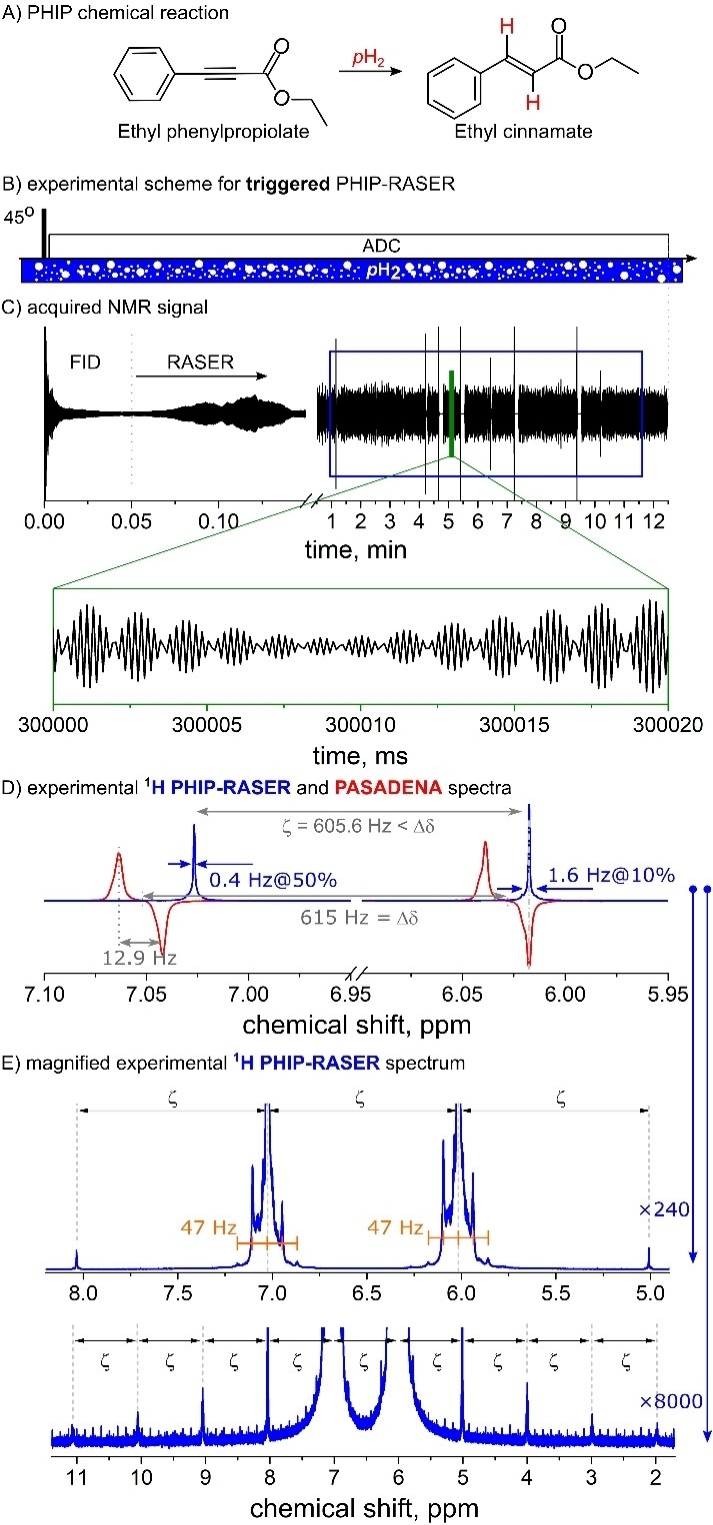
Triggered ^1^H PHIP‐RASER at 600 MHz. An NMR‐RASER was induced by supplying *p*H_2_ into an NMR tube with ethyl phenylpropiolate and Rh‐catalyst (A) coupled to an NMR resonator in situ (B). Upon one 45° RF‐excitation, NMR signal was observed for more than 12 min (C). The Fourier transform of 10.5 min data (C) exhibited two narrow lines with a full width at half maximum of 0.6 ppb (D, E; blue lines: smoothed magnitude spectrum). The right RASER line, which was acquired without frequency lock, was set to match the right negative line of a 45° PASADENA spectrum, acquired with lock and detuned probe (D, red, no radiation damping). The magnified spectrum (E) revealed several types of equidistant satellites, some of them are separated by ζ (E). Similar results were obtained for methyl proprionate (not shown). Details are given in methods.

**Figure 2:**
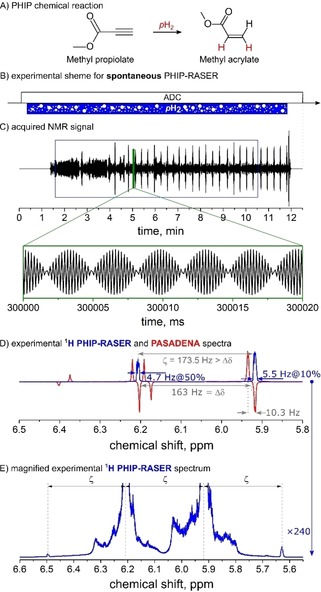
Spontaneous ^1^H PHIP‐RASER at 600 MHz. An NMR‐RASER was induced by supplying *p*H_2_ into an NMR tube with methyl propiolate and Rh‐catalyst (A) coupled to an NMR resonator in situ (B). Without RF‐excitation, spontaneous emission of NMR signal began about one minute after the onset of *p*H_2_ supply and lasted until the *p*H_2_ supply was stopped 11 minutes later (C). The Fourier transform of 9 min data (C) exhibited two narrow lines with a full width at half maximum of 8 ppb (D, E; blue lines: smoothed magnitude spectrum). The RASER signal was acquired without a frequency lock; instead, the right RASER line was aligned to the negative right line in a PASADENA spectrum measured with ^2^H‐lock and detuned probe (D, red). Several but less prominent satellites were observed in a magnified spectrum (E). Similar results were obtained for ethyl phenylpropiolate (not shown). Details are given in methods.

Here, we present a liquid‐state NMR RASER that continuously emits RF‐signal for any given time at room temperature and 600 MHz (Figures 1 and 2) and under the conditions described below. Using parahydrogen‐induced hyperpolarization (PHIP), the RASER begins spontaneously or can be triggered with a standard NMR pulse.

Similar to SABRE‐RASER,^10^ this new effect is based on the spin order of parahydrogen (*p*H_2_) [Eq. (1)],

(1) $${\hat{\rho }}_{pH_{2}}=\frac{\hat{1}}{4}-{\hat{I}}_{1}\cdot {\hat{I}}_{2},$$

which is continuously supplied to a liquid sample (Figures 1B, 2B).^21^

Elegantly, *p*H_2_ has spin 0, hence it does not have magnetization and is impervious to any excitation in its molecular state. Thus, the reservoir of spin order is not affected by pulses or the RASER effect, which takes place in the same physical location.

The production of *p*H_2_ is well established, requiring no more than H_2_ gas flow through a porous catalyst at low temperatures.^22^, ^39^, ^40^ Stored appropriately, *p*H_2_ is stable for days to weeks.^41^ H_2_ can be purchased or produced on‐site chemically or by electrolysis.^42^

In contrast to SABRE‐RASER,^10^ here, *p*H_2_ is permanently incorporated into target molecules via homogeneous hydrogenation (Figures 1A and 2A). The catalytic activity was chosen so that only a small fraction of all precursor molecules is hydrogenated at a time. This way, the reaction was upheld for an extended period of time while PHIP‐RASER occurred.

Here, we demonstrated PHIP‐RASER for two molecules: ethyl cinnamate and methyl acrylate, for our purposes approximately an AX, 2 spin‐1/2 system and an AMX, 3 spin‐1/2 system.

The 600 MHz emission of the RASER began spontaneously approx. 1.5 min after injection of *p*H_2_, or instantaneously after a single “trigger” excitation pulse of 45° (Figures 1 and 2). The “trigger” RF‐pulse converts hyperpolarization into transverse magnetization, which, in turn, induces continuous radiation in a few seconds. The “spontaneous” onset of PHIP‐RASER required a finite hydrogenation time for the accumulation of a hyperpolarized product.

PHIP‐RASER was acquired for a much longer time period than the usual NMR signal. In principal, the duration of the emission (here >10 min) is limited only by *p*H_2_ supply, the hydrogenation catalyst and the reservoir of receiver molecules. In the current implementation, only *p*H_2_ was continuously renewed, but refreshing all constituents can be easily accomplished using a flow setup.^23^, ^24^

After Fourier transformation of ≈10 min RASER signal, two narrow lines were observed at the positions of the added hydrogens, in addition to less pronounced satellites (details of signal processing are given in methods). The line shapes were irregular with a varying full width at half maximum (FWHM) of e. g. ≈0.6 ppb (≈0.4 Hz, Figure 1D) and ≈7.8 ppb (≈4.7 Hz, Figure 2D). The variation in line width may be attributed to varying field homogeneities during *p*H_2_ injection and different spin systems and will have to be investigated elsewhere. Note, that no information about the J‐coupling network in both 2‐ and 3‐spin‐1/2 systems of ethyl cinnamate and methyl acrylate were revealed in the PHIP‐RASER experiments (Figures 1D and 2D).

At the same time, the resonances of the thermally polarized solvent exhibited an inhomogeneously broadened line with FWHM>20 Hz, (Figure S4). Thus it remains unclear, how much of the sample actually emits RASER signal. In the absence of these inhomogeneities and magnetic field drift (no lock was applied), the lower limit for the RASER line width is the nominal spectral resolution of the order of 1 mHz for 11 min. A better homogeneity may be achieved in the future by using more sophisticated techniques to dissolve *p*H_2_ 25, 26, 27 and is currently under investigation. A *p*H_2_ supply that does not disturb the homogeneity of the magnetic field will be instrumental to elucidate PHIP‐RASER effect further (compare Fig 1E and Fig S3).

Importantly, we expect that PHIP‐RASER is functional at all (high) magnetic fields, in parahydrogen and synthesis allow dramatically enhanced nuclear alignment (PASADENA)^28^ – although the spectral appearance depends on the radiation damping rate, polarization level and build‐up rate and may vary (Figs. S3, S9, S10).^13^ PASADENA conditions are met if the J‐coupling between the added hydrogens is weak compared to their chemical shift difference; for J≈10 Hz and a chemical shift difference of 1 ppm, this condition is fulfilled at fields >0.2 T, i. e. at ^1^H frequencies above 10 MHz.

Interestingly, the frequency difference between the RASER lines (ζ ) was found to be different than the nominal chemical shift difference (Δδ ). At the same time, several equidistant satellite resonances with the same spacing ζ were found (Figures 1E and 2E).^13^ The location depends on the radiation damping rate relative to the chemical shift differerence (Figure S8).^13^ Another type of satellite exhibited a more complex structure that strongly depended on the experimental settings. The effect appears to be similar to the spectral clustering effect caused by a distant dipolar field.^29^ The high level of magnetization also causes a drift of the magnetic field and thus a frequency drift of the resonances (Figure S9).^9^, ^29^ All these findings were well reproduced with the density‐matrix based radiation damping theory introduced below.

The interaction between the highly polarized system and RF‐cavity is essential for the RASER emission.^3^, ^4^, ^5^, ^10^, ^11^ This interaction reveals itself as radiation damping. The radiation damping rate is defined as ${\left(\tau_{RD}\right)}^{-1}$ =$\frac{\mu_{0}}{4}\hbar \gamma^{2}\eta Qc_{s}\left|P\right|$ , where $\mu_{0}$ is the vacuum permeability, ℏ is the reduced Planck constant, γ is the gyromagnetic ratio, Q is the quality factor of the coil with a filling factor η , $c_{s}$ is the concentration of the nuclear spin and P is the longitudinal polarization.^30^ This definition of radiation damping rate is very convenient for experiments with large magnetizations in thermal equilibrium, which can be described with modified Bloch equation or, in a more advanced way, with single‐ or multi‐mode LASER/RASER equations.^13^

All experiments in this paper were conducted with a cryogenically cooled coil (Q≈500, see the Supporting Information). The question arises, if the effect can be reproduced with a conventional coil, too. According to the theory of radiation damping, only the filling factor, the Q‐factor of the coil and size of magnetization are important. Thus, it appears plausible that PHIP‐RASER can be observed with room temperature probe with Q > 100, but the experimental proof has to be established. Note that no RASER was observed when continuous SABRE was reported at ≈5 mT, ≈10 % filling factor and a Q≈10‐100.^37^, ^38^

To elucidate this remarkable effect further, we set out to simulate PHIP‐RASER in a coupled two spin‐1/2 system using the density matrix approach and Liouville‐von Neumann equation (LvN, eq S8). It is the most convenient and precise method to simulate liquid state NMR‐experiments with coupled multi‐spin systems.

First of all, it was necessary to introduce an additional element into the conventional liquid state Hamiltonian^30^ to account for radiation damping [Eq. (2)]:^31^

(2) $${\hat{V}}^{rd}\left(t,\hat{\rho }\right)=\omega^{RD}\cdot \sum_{k=1,2}^{\;}{\hat{I}}_{k}=\;=\alpha_{RD}\sum_{k,n=1,2}^{\;}\left(m_{kY}\left(t\right)\;{\hat{I}}_{nX}-m_{kX}\left(t\right){\hat{I}}_{nY}\right)$$

This interaction can be written in analogy to the modified Bloch‐Maxwell equation^32^ and is similar to the multi‐mode RASER equations 17 and 18 from Ref. [13]. Here, $m_{kX,Y}$ =$Tr\left(\hat{\rho }\cdot \frac{1}{2}{\hat{I}}_{kX,Y}^{†}\right)$ /$Tr\left(\frac{1}{2}{\hat{I}}_{kX,Y}\cdot \frac{1}{2}{\hat{I}}_{kX,Y}^{†}\right)$ are the amplitudes of transverse polarization of spin k =1 or 2 of the density matrix $\hat{\rho }$ , the amplitudes in general are time dependent. The magnetic field induced by RD is $\omega^{RD}=\alpha_{RD}\left(\sum_{k=1}^{N\;}m_{kY},-\sum_{k=1}^{N\;}m_{kX}\left(t\right),0\right)$ . The radiation damping rate without polarization factor, $\alpha_{RD}=\frac{\mu_{0}}{4}\hbar \gamma^{2}\eta Qc_{s}$ , is constant if the concentration and electronic circuitry are. On the other hand, ${\left(\tau_{RD}\right)}^{-1}$ depends on the polarization, P, which changes continuously because of relaxation or re‐hyperpolarization.

As initial condition, we assumed a simplified, weakly coupled, two spin‐1/2 system after hydrogenation with *p*H_2_ [Eq. (1)] as [Eq. (3)]:^21^

(3) $${\hat{\rho }}_{PASADENA}=\frac{\hat{1}}{4}-{\hat{I}}_{1Z}{\hat{I}}_{2Z}$$

The supply of para‐order to the system was implemented with a source operator [Eq. (4)]:

(4) $$\hat{S}=-W_{in}\left(t\right)\left({\hat{I}}_{1}\cdot {\hat{I}}_{2}\right)$$

No unity operator was used [see Equation (1)] to keep the trace over the density matrix equal to 1. $W_{in}\left(t\right)$ is a time‐dependent rate of the polarization influx. Depending on the simulated experiment, different source operators can be used. Simulations were performed using the MOIN spin library; the source code is available online.^33^, ^34^

The addition of RD into the LvN equation makes the simulation of polarization transfer in the presence of RD^35^ straightforward. Using the published SABRE models including chemical exchange, the same approach can be used to simulate SABRE‐RASER.^33^, ^36^ A more detailed description of the theory and more simulation examples are given in SI. The conditions of the RASER threshold are discussed in more detail in Ref. [13].

**Figure 3:**
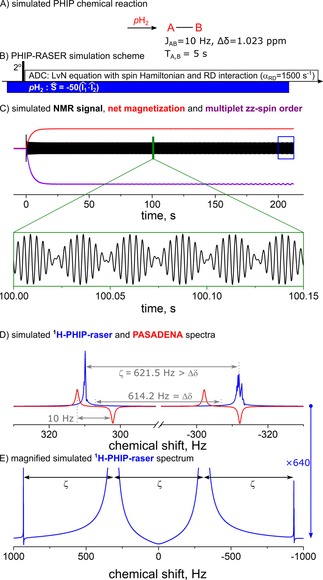
Simulation of ^1^H PHIP‐RASER at 600 MHz. A two spin‐1/2 system was continuously hyperpolarized by means of PHIP (A, J = 10 Hz, Δδ = 1.023 ppm, relaxation times 5 s). The evolution of the system after 2° RF excitation pulse was calculated using the LvN equation (C, $B_{0}$ =14.1 T, $\alpha_{RD}$ =1500 s^−1^ and $W_{in}$ =50 s^−1^). Radiation damping was found to induce zz‐spin order (C, violet), longitudinal (m_1Z_, red) and transverse magnetization, the RASER signal (m_1X_+m_2X_, black line, with magnified view). The last 10 s of the transverse magnetization (C, blue square) were Fourier transformed and exhibited two lines (D, E; blue lines; magnitude, apodized with exp(‐t/2s)). A simulated PASADENA spectrum was added for reference (D, red line, no radiation damping, FWHM = 1 Hz). Several equidistant but smaller satellites at ζ were found in the enlarged RASER spectrum (E, compare with Fig 1E,2E). The right RASER resonance was shifted by +18 Hz to match the negative right line in PASADENA spectrum.

With these additions [Equations (2)–(4)], we were able to reproduce all essential PHIP‐RASER features (Figure 3 and the Supporting Information): (i) two PHIP‐RASER lines (Figure 3D), (ii) a distance between the RASER lines ζ that is not equal to the chemical shift difference Δδ and which depends on RD parameters (Figure 3D and Figure S9), (iii) an equidistant frequency‐comb (Figure 3E) and (iv) an overall frequency shift of the resonances (the latter was also reported in Refs. [9, 29] Fig. S9).

We foundnd that the experimental signal exhibits much stronger amplitude variations than the simulated one. We attribute this effect to the magnetic field inhomogeneity induced by the *p*H_2_ supply, which was not considered in the simulations.

The same simulations were used to shed light into the classic PASADENA experiment, too. For example, an asymmetric line broadening and emission of “spontaneous”, echo‐like RASER bursts were observed up to 30 s after the *p*H_2_ supply was stopped (see Figs. S7, S11). Simulations revealed that radiation damping results in conversion of PASADENA spin order (eq 3) into longitudinal $m_{z}-$ and transversal $m_{xy}-$ magnetization (Figs. S10 and S11). Experimentally, this is manifested in an asymmetric broadening of spectral lines (Fig, S10, SI) and RASER bursts, which may confound the interpretation of the spectra or cause inefficient polarization transfer to heteronuclei. The effect of RD on ^1^H‐PASADENA spectra and on the efficiency of polarization transfer to ^13^C was recently and experimentally demonstrated by Korchak et al.^35^ at room temperature at fields of 1 T and 7 T.

## 3 Conclusions

These findings establish PHIP‐RASER as the first, long‐lasting ^1^H NMR‐RASER at high field (and high frequencies), operating at room temperature and in the liquid state. Using standard commercial equipment, the method can be easily implemented in any NMR laboratory. The key element is a strong coupling between the resonator and hyperpolarized sample.

With a frequency range of 10 MHz and above, PHIP‐RASER is very flexible. The maximum frequency is currently limited only by the static magnetic fields available (≈1.2 GHz).

The long emission >10 min allows to partially negate the detrimental effects of the strong inhomogeneity induced by *p*H_2_ supply, as narrow lines of <1 ppb were observed.

Given a more homogeneous delivery of *p*H_2,_ it appears feasible to further improve on these linewidths. Simulations suggest that extremely narrow lines of a few mHz can be obtained at 600 MHz, effectively overcoming the T_2_ limit. Note that at the same time, the full, 14‐T‐chemical shift dispersion is maintained. These and other applications yet to discover and implement (like RASER gyroscopes^13^) make PHIP‐RASER a highly interesting effect for NMR, physics, chemistry and beyond.

## Experimental Section

### Materials

The sample solution contained 4 mM 1,4‐Bis(diphenylphosphino) butane (1,5‐cyclooctadiene) rhodium(I) tetrafluoroborate (Strem Chemicals, CAS: 79255‐71‐3) and 60 mM ethyl phenylpropiolate (EP, Figure 1A, Sigma‐Aldrich, CAS: 2216‐94‐6) or 100 mM methyl propiolate (MP, Figure 2A, Sigma‐Aldrich, CAS: 922‐67‐8) dissolved in acetone‐d_6_ 99.8 % (Deutero GmbH, CAS: 666‐52‐4). Upon hydrogenation, ethyl cinnamate (EC, Figure 1A) or methyl acrylate (MA, Figure 2A) was formed.

### Experimental Setup

Experiments were carried out on a 600 MHz spectrometer (Bruker Avance II) with a cryogenically cooled probe (TCI) with Q=v/Δv≅ 600.2 MHz/1.2 MHz≅500 (see SI, Fig. S3) and 5 mm screw‐cap NMR tubes (Wilmad). Tubes were filled with 500 μl of the sample solution. *p*H_2_ was prepared using a home‐build liquid nitrogen generator that provides a 50 % para‐hydrogen. The gas was delivered into the spectrometer by a 1/16′′ polytetrafluoroethylene (PTFE) capillary. A hollow optical fibre was glued with epoxy resin to the end of the capillary and inserted into the NMR tube and solution (Molex, part. num. 106815‐0026, internal diameter 250 μm and outer diameter 360 μm). A *p*H_2_ pressure of approx. 1.2 bar (0.2 bar overpressure to atmosphere) was used to achieve a steady bubbling.

### Protocol 1 (Scheme 1)

*p*H_2_ was supplied (bubbled) into the sample solution for $\tau_{b}$ =10 s to hydrogenate EP (or MP) and generate PASADENA. After that, an optional rectangular, 4 μs 45° RF‐pulse was applied (optional). All experiments were carried out at 25 °C and ambient pressure. During the experiments, some convection and diffusion occurred. *p*H_2_ bubbling was stopped only after approx. 12 minutes of signal acquisition. Unfortunately, the NMR spectrometer did not allow for continuous data acquisition. Instead, the data was saved in 9 (Figure 1B) or 8 (Figure 2B) blocks of 90 s and 1730768 points each. The blocks were acquired sequentially with a 1–3 s delay in between, required to load and start the new acquisition. For the figures, the spectra of the first and last block in each set were omitted to exclude transient effects at the onset and end of the *p*H_2_ injection. Magnitude RASER spectra were smoothed after averaging using a Savitzky‐Golay filter with first‐order polynomial for a window of 50 points (∼0.22 Hz or °0.36 ppb). Post‐processing methods to improve magnetic field stability / homogeneity as in Ref. [13] e.g. were not applied.

**Scheme 1:**
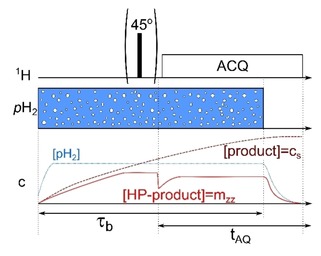
Scheme of the experimental workflow (Protocol 1): i) At a given time, the *p*H_2_ supply is initiated and upheld for a period $\tau_{b}$ . ii) optional excitation by hard 45^o^ RF pulse to “trigger” RASER, and iii), signal acquisition (ACQ) in multiple blocks of 90 s each. Below, the concentrations of *p*H_2_ (dotted blue line), total product ([product]=c_s_, dashed wine line) and hyperpolarized product ([HP‐product]=m_zz_, solid red line) are plotted qualitatively. Note that *p*H_2_ is immune to excitations with RF pulses.

### Simulation

Simulation parameters for Figure 3: initial density matrix $\hat{\rho }\left(-0\right)=\hat{1}/4-{\hat{I}}_{1Z}{\hat{I}}_{2Z}$ , equilibrium state ${\hat{\rho }}_{eq}=\hat{1}/4$ , J=10 Hz, chemical shift difference 1.014 ppm, B_0_=14.1 T, $\alpha_{RD}$ =1500 s^−1^, relaxation time 5 s and rate of polarization influx $W_{in}=50\;\;s$ ^−1^. The simulation model is described in SI and source code is available online.^34^

### Data Availability

All data is available from the corresponding authors upon reasonable request.

## Supporting information

As a service to our authors and readers, this journal provides supporting information supplied by the authors. Such materials are peer reviewed and may be re‐organized for online delivery, but are not copy‐edited or typeset. Technical support issues arising from supporting information (other than missing files) should be addressed to the authors.

Supplementary
