# Supplementary material for: Continuous Radio Amplification by Stimulated Emission of Radiation using Parahydrogen Induced Polarization (PHIP‐RASER) at 14 Tesla
Source: Chemphyschem. 2020 Feb 11;21(7):667–72. doi: 10.1002/cphc.201901056 (PMC7187451; doi:10.1002/cphc.201901056)
Supplement: Supplementary file 1 — Supplementary [file CPHC-21-667-s001.pdf]

CHEMPHYSCHEM

Supporting Information

Continuous Radio Amplification by Stimulated Emission of Radiation using Parahydrogen Induced Polarization (PHIP-RASER) at 14 Tesla

Andrey N. Pravdivtsev,* Frank D. Sönnichsen, and Jan-Bernd Hövener*© 2020 The Authors. Published by Wiley-VCH Verlag GmbH & Co. KGaA.
This is an open access article under the terms of the Creative Commons Attribution License, which permits use, distribution and reproduction in any medium, provided the original work is properly cited.

Table of Contents

Table of Contents 2

1. Evolution of PHIP-RASER emission (supplementary to Fig 1). Repetition of the experiment on another day 3

2. Evolution of PHIP-RASER emission (supplementary to Fig S1) 4

3. Evolution of PHIP-RASER emission (enlarged Fig S1) 5

4. NMR spectra acquired during and without pH2 bubbling 6

5. WOBB image of used NMR probe 7

6. Calculation of radiation damping time and other related parameters 8

7. Radiation damping model 9

8. Simulation: radiation damping in the case of thermal equilibrium 11

9. Simulation: spontaneous RASER emission in PASADENA experiment. Production of hyperpolarization is represented by superposition of delta functions . 12

10. Simulation: RASER emission as a result of an influx of negative net magnetization 13

11. Simulation: PHIP-RASER spectra as a function of radiation damping amplitude 14

12. Experimental protocol 2: stop bubbling scheme 15

13. Simulation: effect of radiation damping on PASADENA 16

14. Spontaneous PHIP-RASER bursts (protocol 2) 17

References 19

1. **Evolution of PHIP-RASER emission (supplementary to Fig 1). Repetition of the experiment on another day.**

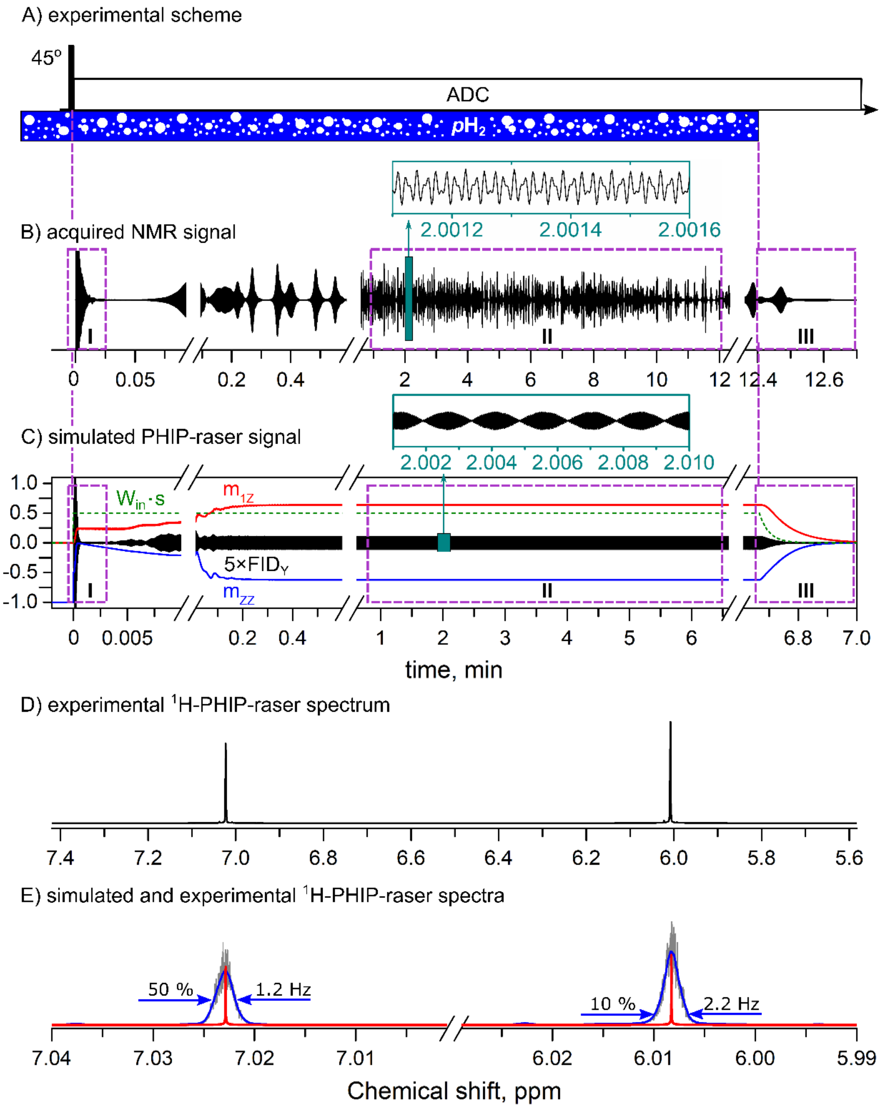

Figure S1: 12 min of PHIP-RASER emission of ^{1}H NMR signal at 600 MHz. A liquid state NMR-RASER was induced by supplying *p*H_2 into an NMR tube coupled to a resonator in situ (A). Upon one 45° RF-excitation, NMR signal was observed for more than 12 min until the *p*H_2 supply was stopped (B). The data was well reproduced by a newly developed quantum mechanical model (C). We found that PASADENA polarization (m_{ZZ}) is converted into longitudinal (m_Z) and transverse (m_Y) magnetization by the coupling to the resonator. RASER spectrum exhibited two narrow lines with a full width at half maximum of 2 ppb (D, E; black lines: experimental magnitude spectrum, blue: smoothed experimental spectrum, red: simulated spectrum with 6 mHz line broadening). Inserts on B and C demonstrate the presence of these two frequencies in the signal (frequencies of the rotating frame of reference were different). PHIP-RASER spectrum (D) is average of 10 magnitude spectra acquired with an interval of 3-5 s that corresponds to 10 FIDs with a duration of 1 minute (dashed block II on B). No apodization was applied.

2. Evolution of PHIP-RASER emission (supplementary to Fig S1)

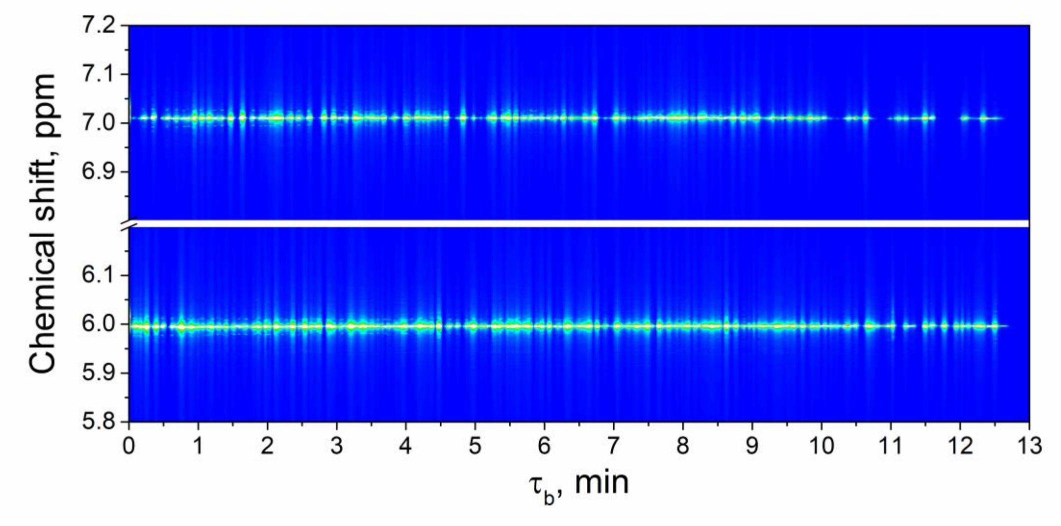

Figure S2. Observed evolution of two PHIP-RASER active lines (supplementary to Fig S1B). Whole FID (13 minutes) was combined from 13 FIDs with a duration of 1 minute acquired with an interval of 3-5 s. Whole FID was divided into blocks with a duration of AQ=2 s, then Fourier transformation (FT) to each block was applied. Note that field inhomogeneity and convection due to $p\mathrm{H}_2$ bubbling did not allow continuous stable RF-emission.

3. Evolution of PHIP-RASER emission (enlarged Fig S1)

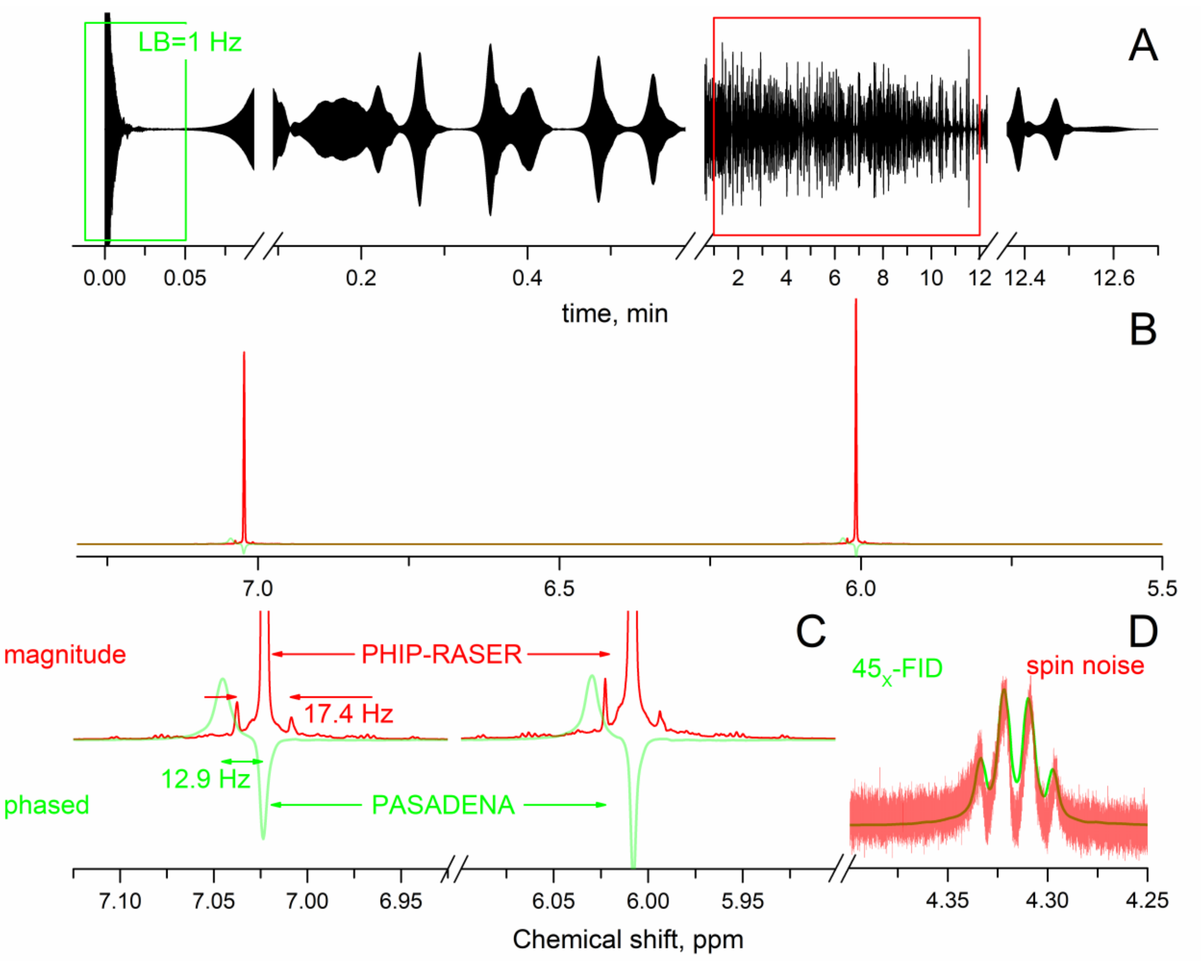

Figure S3: 12 min of RASER emission of ^{1}H NMR signal at 600 MHz. A liquid state NMR-RASER was induced by supplying pH$_2$ into an NMR tube coupled to a resonator in situ. Upon one 45° RF-excitation, NMR signal was observed for more than 12 min until the pH$_2$ supply was stopped (A). The Fourier transform of 11 min data (A: red box) exhibited two narrow lines with a full width at half maximum of 2 ppb (B, C: red, magnitude). The Fourier transform of the first 3 seconds (A: green box; 1 Hz line broadening is applied) exhibited classical PASADENA pattern (B, C: green, phased). Example of spin noise acquired spectrum (D: red, magnitude) and comparison with thermal spectrum (D: green, phased). PASADENA spectral line intensities are affected by RD effect (C). Amplitudes of spectra were adjusted for better visualization of results. Smaller satellite frequency splitting than on Fig 1 indicates that in this case achieved radiation damping amplitude was smaller.

4. NMR spectra acquired during and without pH2 bubbling

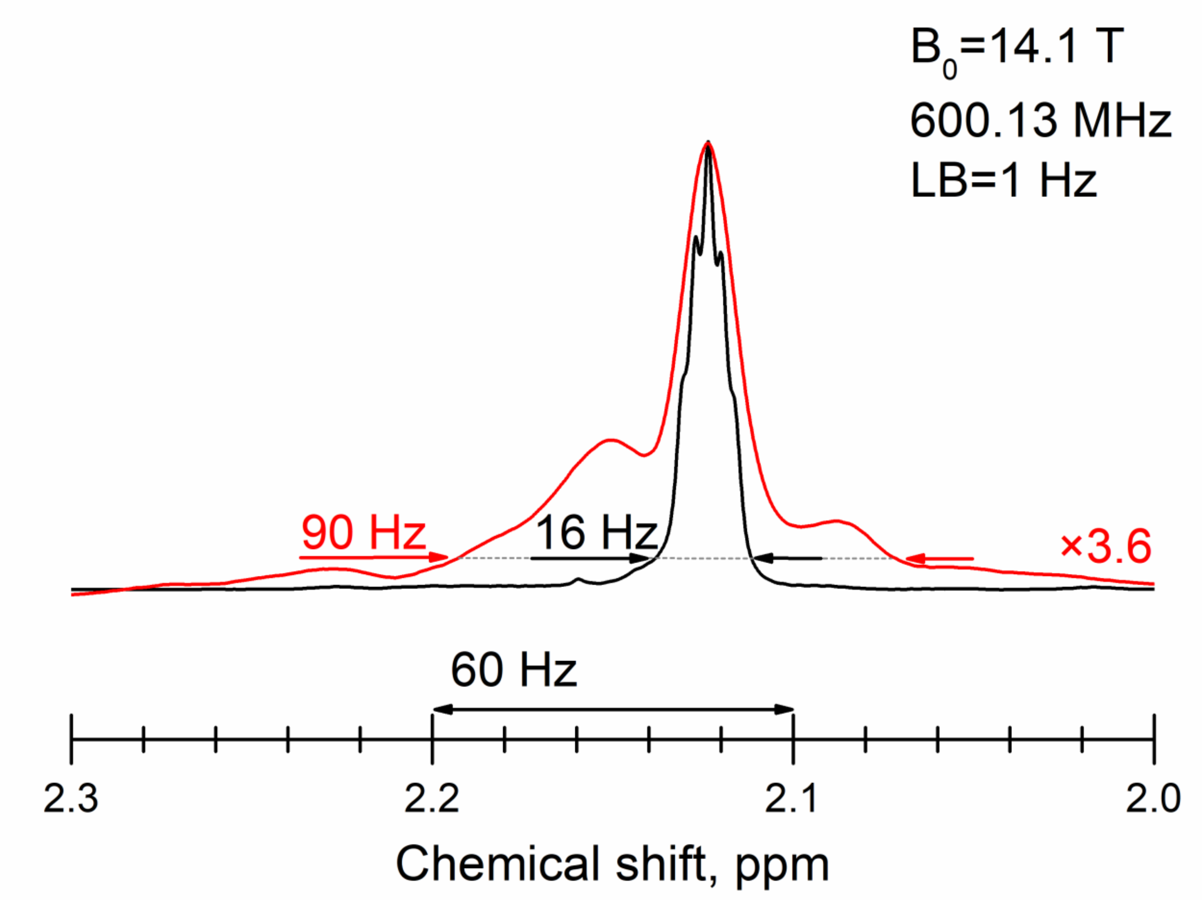

Figure S4. Homogeneous NMR spectrum (black) and spectrum acquired when *p*H_2 bubbling was on (red). Applied line broadening was 1 Hz. FID of the “red” spectrum is shown on Fig. S1:B (dashed box I).

5. WOBB image of used NMR probe

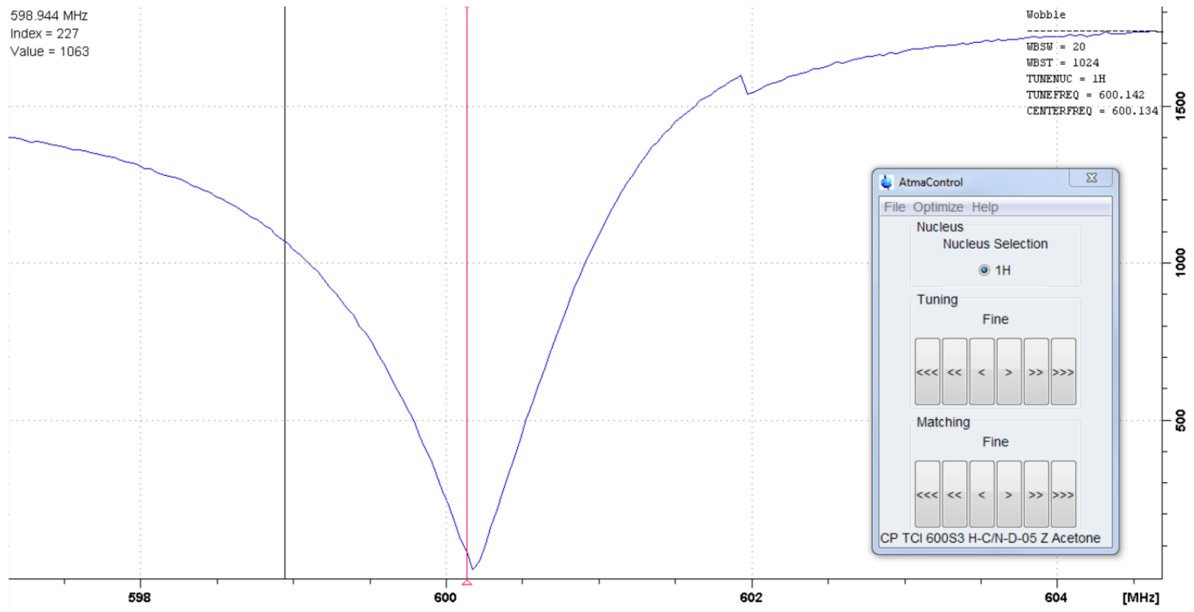

Figure S5. Screenshot of a WOBB window in TopSpin for the used cryogenically cooled probe (TCI). It follows that Q=$\nu_0/\Delta\nu_{1/2}$ ≅600 MHz / 1.2 MHz ≅ 500.

6. Calculation of radiation damping time and other related parameters

Thermal polarization level and magnetization of spin-½ are given by[1]:

$$P = \tanh\left(\frac{\hbar\gamma B_0}{2k_B T}\right) \cong \frac{\hbar\gamma}{2k_B T} B_0 \ll 1 \qquad \text{(eq S1)}$$

and

$$M_0 = \frac{1}{2}\hbar\gamma n_s |P| \cong n_s \frac{\hbar^2\gamma^2}{4k_B T} B_0 \qquad \text{(eq S2)}$$

Then radiation damping rate, $(\tau_{RD})^{-1}$=$\alpha_{RD}|P|$ and parameter α_{RD} are given by the following equations[1]:

$$(\tau_{RD})^{-1} = \frac{1}{2}\mu_0 \eta Q |\gamma M_0| = \frac{\mu_0}{4}\hbar\gamma^2 \eta Q c_s (|P| \cdot f) = \frac{\mu_0}{8k_B T}\eta Q n_s \hbar^2 \gamma^3 B_0 \qquad \text{(eq S3)}$$

$$\alpha_{RD} = (\tau_{RD})^{-1}/m = \frac{\mu_0}{4}\hbar\gamma^2 \eta Q c_s \qquad \text{(eq S4)}$$

Here $m = |P| \cdot f$, where f is a fraction of c_s (the rest is assumed to be not polarized) polarized to level P. In case of thermal polarization or when average level of polarization is used $f = 1$. In PHIP, f reflects the fraction of the reagent with concentration c_s currently hyperpolarized.
Filling factor, η is usually in the range of 0.1-1 and is defined as follows[1]:

$$\eta \cong \frac{\text{volume of sample}}{\text{volume of coil}} \qquad \text{(eq S5)}$$

Below we will calculate the value of P, M_0 and $(\tau_{RD})^{-1}$ and α_{RD} for H_2O at 300 K temperature and 10 Tesla magnetic field. High product ηQ = 100 is assumed.
All necessary constants and values:

$\gamma_{1H} \cong 267.5 \cdot 10^6 \ \frac{rad}{s \cdot T}$ – gyromagnetic ratio of ^{1}H nuclei spin,

$\hbar \cong 1.054 \cdot 10^{-34} \ \frac{m^2 \cdot kg}{rad \cdot s}$ – Plank constant,

$c_s(H_2O) = 2c(H_2O) \cong 6.7 \cdot 10^{28} \ \frac{1}{m^3} = 6.7 \cdot 10^{22} \ \frac{1}{cm^3}$ – concentration of ^{1}H in pure ^1H_2O,

$\mu_0 = 4\pi \cdot 10^{-7} \ \frac{T \cdot m}{A} \cong 1.257 \cdot 10^{-6} \ \frac{T \cdot m}{A}$ – vacuum permeability,

$k_B \cong 1.38 \cdot 10^{-23} \ \frac{m^2 \cdot kg}{s^2 \cdot K}$ – Boltzmann constant,

$[T] = \frac{kg}{A \cdot s^2}$ – Tesla units in the international system of units (known as SI).

Note that here we are keeping radian units for convenience.
Thermal polarization of ^{1}H at 300 K and 10 T:

$$P \cong \frac{\hbar\gamma B_0}{2k_B T} \cong \frac{1.054 \cdot 10^{-34} \cdot 267.5 \cdot 10^6 \cdot 10 \cdot \frac{m^2 \cdot kg}{rad \cdot s} \cdot \frac{rad}{s \cdot T} \cdot T}{2 \cdot 1.38 \cdot 10^{-23} \cdot 300 \ \frac{m^2 \cdot kg}{s^2 \cdot K} \cdot K} = \frac{1.054 \cdot 10^{-27} \cdot 267.5}{2.76 \cdot 10^{-23} \cdot 300} \cong 3.4 \cdot 10^{-5}.$$

The thermal magnetization of ^{1}H of ^1H_2O at 300 K and 10 T:

$$M_0 = \frac{1}{2}\hbar\gamma n_s P \cong \frac{1}{2} 1.054 \cdot 10^{-34} \cdot 267.5 \cdot 10^6 \cdot 6.7 \cdot 10^{28} \cdot 3.4 \cdot 10^{-5} \ \frac{m^2 \cdot kg}{rad \cdot s} \cdot \frac{rad}{s \cdot T} \cdot \frac{1}{m^3} \cong 0.0322 \ \frac{kg}{s^2 \cdot m \cdot T}.$$

Radiation damping rate of ^{1}H of ^1H_2O at 300 K and 10 T:

$$(\tau_{RD})^{-1} = \frac{1}{2}\mu_0 \eta Q |\gamma M_0| \cong \frac{1}{2} 1.257 \cdot 10^{-6} \cdot 100 \cdot 267.5 \cdot 10^6 \cdot 0.0322 \ \frac{T \cdot m}{A} \cdot \frac{rad}{s \cdot T} \cdot \frac{kg}{s^2 \cdot m \cdot T} =$$

$$= \frac{1}{2} 1.257 \cdot 2.675 \cdot 0.0322 \cdot 10^4 \ \frac{kg \cdot rad}{A \cdot s^3 \cdot T} \cong 540 \ \frac{rad}{s},$$

and hence $\tau_{RD} \cong 1.8 \, ms$,
Parameter α_{RD} for ^{1}H of ^1H_2O at 300 K and 10 T:

$$\alpha_{RD} = \frac{\mu_0}{4}\hbar\gamma^2 \eta Q n_s = \frac{(\tau_{RD})^{-1}}{P} = \frac{540}{3.4 \cdot 10^{-5}} \ \frac{rad}{s} \cong 1.6 \cdot 10^7 \ \frac{rad}{s}$$

Hence all together: $P \cong 3.4 \cdot 10^{-5}$, $M_0 \cong 0.0322 \ \frac{kg}{s^2 \cdot m \cdot T}$, $(\tau_{RD})^{-1} \cong 540 \ \frac{rad}{s}$, $\tau_{RD} \cong 1.8 \, ms$ and $\alpha_{RD} \cong 1.6 \cdot 10^7 \ \frac{rad}{s}$ for ^{1}H of ^1H_2O at 300 K, 10 T and $\eta Q = 100$.

7. Radiation damping model

To describe the RD of a single nucleus, it is convenient to use a modified Bloch-Maxwell equation proposed by Bloembergen and Pound that in the rotating frame of reference can be written as[2]

$$\frac{d}{dt}\begin{pmatrix} M_X \\ M_Y \\ M_Z \end{pmatrix} = \begin{pmatrix} -R_2 & \omega_Z & -\omega_Y \\ -\omega_Z & -R_2 & \omega_X \\ \omega_Y & -\omega_X & -R_1 \end{pmatrix}\begin{pmatrix} M_X \\ M_Y \\ M_Z \end{pmatrix} + \frac{1}{M_0\tau_d}\begin{pmatrix} -M_X M_Z \\ -M_Y M_Z \\ M_X^2 + M_Y^2 \end{pmatrix} + \begin{pmatrix} 0 \\ 0 \\ R_1 M_0 \end{pmatrix} \qquad \text{(eq S6a)}$$

M_X, M_Y, M_Z are the components of magnetization vector $\boldsymbol{M}$ with Z being along magnetic field B_0. $M_0 = \frac{1}{2}\hbar\gamma c_s|P|$ is a value of equilibrium magnetization $\boldsymbol{M_0} = (0,0,M_0)$. $\hbar$ is the reduced Planck constant. γ is a gyromagnetic ratio. c_s is a nuclei spin concentration and P is the value of longitudinal polarization. $(\tau_d)^{-1} = \frac{\mu_0}{2}\eta Q|\gamma M_0|$ is the classical radiation damping rate,[1] where μ_0 is a vacuum permeability, Q is the quality factor of the coil with the filling factor η. It can be rewrite as follows:

$$\frac{d}{dt}\begin{pmatrix} M_X \\ M_Y \\ M_Z \end{pmatrix} = \begin{pmatrix} 0 & \omega_Z & -\omega_Y \\ -\omega_Z & 0 & \omega_X \\ \omega_Y & -\omega_X & 0 \end{pmatrix}\begin{pmatrix} M_X \\ M_Y \\ M_Z \end{pmatrix} - \begin{pmatrix} R_2 & 0 & 0 \\ 0 & R_2 & 0 \\ 0 & 0 & R_1 \end{pmatrix}\begin{pmatrix} M_X \\ M_Y \\ M_Z - M_0 \end{pmatrix} + \frac{1}{M_0\tau_d}\begin{pmatrix} 0 & 0 & -M_X \\ 0 & 0 & -M_Y \\ M_X & M_Y & 0 \end{pmatrix}\begin{pmatrix} M_X \\ M_Y \\ M_Z \end{pmatrix} \qquad \text{(eq S6b)}$$

Using vector notations: $\boldsymbol{M}$, $\boldsymbol{M_0}$, $\boldsymbol{R}$ and $\boldsymbol{\omega}$ and vector product, $[\cdot \times \cdot]$, eq S6b can be simplified as

$$\frac{d\mathbf{M}}{dt} = [\boldsymbol{M} \times \boldsymbol{\omega}] - \boldsymbol{R}(\boldsymbol{M} - \boldsymbol{M_0}) + \frac{1}{M_0\tau_d}[\boldsymbol{M} \times (-M_Y, M_X, 0)] \qquad \text{(eq S7a)}$$

or

$$\frac{d\mathbf{M}}{dt} = \left[\boldsymbol{M} \times \boldsymbol{\omega^{effRD}}(\boldsymbol{M_X}, \boldsymbol{M_Y})\right] - \boldsymbol{R}(\boldsymbol{M} - \boldsymbol{M_0}) \qquad \text{(eq S7b)}$$

With an effective magnetic field, $\boldsymbol{\omega^{effRD}} = \boldsymbol{\omega} + \frac{1}{M_0\tau_d}(-M_Y, M_X, 0)$, that includes applied magnetic fields, $\boldsymbol{\omega}$, and radiation damping magnetic field, $\boldsymbol{\omega^{RD}} = \frac{1}{M_0\tau_d}(-M_Y, M_X, 0)$.

While Bloch equations are well suited to describe a single-spin system, more complex effects are not included. To describe coupled multi-spin systems, it is convenient to use Liouville-von Neumann equation (LvN). However, a LvN equation that takes into account RD effect was not found in the literature.

Using eq S7b, an addition of RD effect to LvN equation is straightforward because it is simply interaction of spin with an "external" magnetic field[3]:

$$\frac{d}{dt}\widehat{\boldsymbol{\rho}}(t) = -i\left(\widehat{\widehat{\mathrm{H}}} + \widehat{\widehat{\mathrm{V}}}^{rd}(t, \widehat{\boldsymbol{\rho}})\right)\widehat{\boldsymbol{\rho}}(t) + \widehat{\widehat{R}}\left(\widehat{\boldsymbol{\rho}}(t) - \widehat{\boldsymbol{\rho}}_{eq}\right) + \widehat{\boldsymbol{S}} \qquad \text{(eq S8)}$$

Where $\widehat{\boldsymbol{\rho}}$ is a density matrix, $\widehat{\widehat{R}}$ is a relaxation superoperator, $\widehat{\boldsymbol{\rho}}_{eq}$ is an equilibrium density matrix, $\widehat{\widehat{\mathrm{H}}} = [\widehat{\mathrm{H}}, \cdot]$ is Hamiltonian superoperator of Hamiltonian $\widehat{\mathrm{H}}$, and $\widehat{\widehat{\mathrm{V}}}^{rd} = [\widehat{\mathrm{V}}^{rd}, \cdot]$ is a RD superoperator of RD operator $\widehat{\mathrm{V}}^{rd}$. $\widehat{\boldsymbol{S}}$ is a source of hyperpolarization (see below). For calculating relaxation superoperators $\widehat{\widehat{R}}$, we used a local fluctuating magnetic fields relaxation model, where relaxation of individual spin is defined by a single relaxation time at high magnetic field[4–6]. Then for the multi-spin system in the liquid state Hamiltonian, $\widehat{\mathrm{H}}$, and relaxation damping interaction, $\widehat{\mathrm{V}}^{rd}$, are

$$\widehat{\mathrm{H}} = -\sum_{k=1}^{N}\boldsymbol{\omega}_k \cdot \hat{\mathbf{I}}_k + 2\pi\sum_{1 \le k < m \le N} J_{km}\hat{\mathrm{I}}_{kZ}\hat{\mathrm{I}}_{mZ} \qquad \text{(eq S9)}$$

$$\widehat{\mathrm{V}}^{rd}(t, \widehat{\boldsymbol{\rho}}) = \boldsymbol{\omega^{RD}} \cdot \sum_k \hat{\mathbf{I}}_k = \alpha_{RD}\sum_{k,n=1}^{N}(m_{kY}(t)\,\hat{\mathrm{I}}_{nX} - m_{kX}(t)\hat{\mathrm{I}}_{nY}) \qquad \text{(eq S10) = (eq 2)}$$

Here $m_{kX,Y} = Tr\left(\widehat{\boldsymbol{\rho}} \cdot \frac{1}{2}\hat{I}_{kX,Y}^{\dagger}\right) / Tr\left(\frac{1}{2}\hat{I}_{kX,Y} \cdot \frac{1}{2}\hat{I}_{kX,Y}^{\dagger}\right)$ are amplitudes of polarization of $\hat{I}_{kX}$ and $\hat{I}_{kY}$ components of spin k of the density matrix $\widehat{\boldsymbol{\rho}}$, the amplitudes, in general, are time-dependent. In the main text we also showed evolution of polarization of two-spin longitudinal spin order calculated as $m_{ZZ} = Tr(\widehat{\boldsymbol{\rho}} \cdot \hat{I}_{1Z}\hat{I}_{2Z}) / Tr(\hat{I}_{1Z}\hat{I}_{2Z} \cdot \hat{I}_{1Z}\hat{I}_{2Z})$. $\alpha_{RD} = \frac{\mu_0}{4}\hbar\gamma^2\eta Q c_s = (\tau_{RD})^{-1}/|P|$ is a radiation damping rate without polarization factor. $\widehat{\mathrm{V}}^{rd}$ can be understood in the same way as interaction

of RD induced magnetic field vector $\boldsymbol{\omega^{RD}} = \frac{1}{M_0 \tau_d}(-M_Y, M_X, 0)$ with a magnetization (eq S7 and S10). For multi-mode RASER, RD is induced by all polarized spins:

$$\boldsymbol{\omega^{RD}} = \alpha_{RD}\left(\sum_{k=1}^{N} m_{kY}\,, -\sum_{k=1}^{N} m_{kX}(t)\,, 0\right)$$

RD operator, $\hat{V}^{rd}$, is similar to interactions in multi-mode RASER equation 17[7] applied to the whole density matrix.
During hyperpolarization experiments, magnetization can change due to:

- variation of concentration of the hyperpolarized agent, c_S, and
- variation of the level of average polarization, $|P|$, due to e.g., relaxation and RF-excitation.

The rate of RD is linearly proportional to both parameters, which were not distinguished in the simulations; their product was represented by the corresponding amplitudes, $m_{kX,Y}$, of the density matrix.
For the protons of water, with a concentration of ~110 M and a $\eta Q \cong 100$, $\alpha_{RD} = \frac{\mu_0}{4}\hbar\gamma^2\eta Q c_s \cong 10^7$ s^{-1}, in thermal equilibrium at 9.4 T P(^{1}H) $\cong 3.4 \cdot 10^{-5}$ so that $(\tau_{RD})^{-1} \cong 540$ s^{-1} or $\tau_{RD} \cong$ 1.8 ms (see evaluation in the previous section): this is an example of a very strong RD effect in thermal equilibrium. However, when the concentration is reduced by 5 orders of magnitude to 1 mM, then $\alpha_{RD} \cong 10^3$ s^{-1} and at thermal equilibrium $\tau_{RD} \cong 180\,s \gg T_2^*$ hence RD can be completely neglected. On the other hand, when 1 mM sample is hyperpolarized to ~100 %, $\tau_{RD} \cong 6$ ms and radiation damping effect already play a crucial role.
The source of hyperpolarization, $\hat{\boldsymbol{S}}$, was introduced into eq. S8 to mimic PHIP experimental conditions. A similar approach was used before e.g., to model SABRE[8,9] and DNP[10]. During the experiment, the hydrogenation reaction carries on, corresponding to an influx of para spin order into the spin system. For a two spin-1/2 system and enrichment of pH$_2$ to 100 %, the source operator can be written as

$$\hat{\boldsymbol{S}} = W_{in}(t)\left[-\left(\hat{\mathbf{I}}_1 \cdot \hat{\mathbf{I}}_2\right)\right] \quad \text{(eq S11) = (eq 4)}$$

Where $-\left(\hat{\mathbf{I}}_1 \cdot \hat{\mathbf{I}}_2\right)$ is the traceless part of the pH$_2$ density matrix, and W_{in} is the rate of hyperpolarization income (generally time-dependent). We used a constant value and an exponential decay function (Fig 3, S1 and S11) and a superposition of delta functions (Fig S7) to construct W_{in}(t). Depending on the simulated experiment, different source operator was used (Fig S7, S8). Simulations were performed using the MOIN spin library[9,11]; the source code is available online.

8. Simulation: radiation damping in the case of complete polarization in a weekly coupled two spin system

To test introduced above RD model, we simulated completely polarized, weakly coupled two spin-½ system. The simulated RD effect was similar to that observed experimentally: transversal and longitudinal magnetization were depleted or restored faster, respectively (Fig. S6B). All spectral components were equally broadened (Fig. S6D).
These RD effects are well known,[12] but calculated for the coupled multi-spin system using density matrix approach for the first time. In the same way, RD effects can be simulated for any NMR pulse sequences and coupled multi-spin systems.

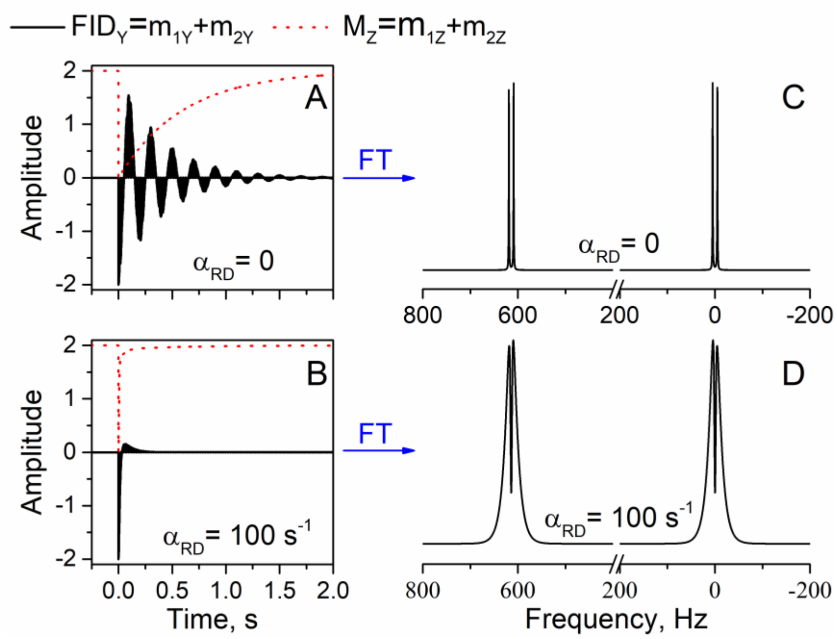

Figure S6. Simulated free induction decay (FID$_Y$=$\mathrm{m}_{1Y}+\mathrm{m}_{2Y}$), longitudinal magnetization ($\mathrm{M}_Z = m_{1Z}+\mathrm{m}_{2Z}$) (A, B) and corresponding NMR spectra (C, D) for an AX spin system with RD (B, D: α_{RD}=100 s^{-1}) and without RD (A, C: α_{RD}=0). A completely polarized system, $\hat{\rho}(-0) = \hat{\rho}_{eq} = \hat{1}/4 + (\hat{I}_{1Z}+\hat{I}_{2Z})/2$, was assumed. At time point 0 ideal 90^{o}_{-X} rotation pulse was applied. Simulation parameters: B_0=14.1 T, J = 10 Hz, W_{in}=0, chemical shift difference 1.023 ppm and relaxation time 0.6 s.

9. Simulation: spontaneous RASER emission in PASADENA experiment. Production of hyperpolarization is represented by superposition of delta functions

This simulation shows that after the introduction of polarization the system needs some time before it starts to emit.

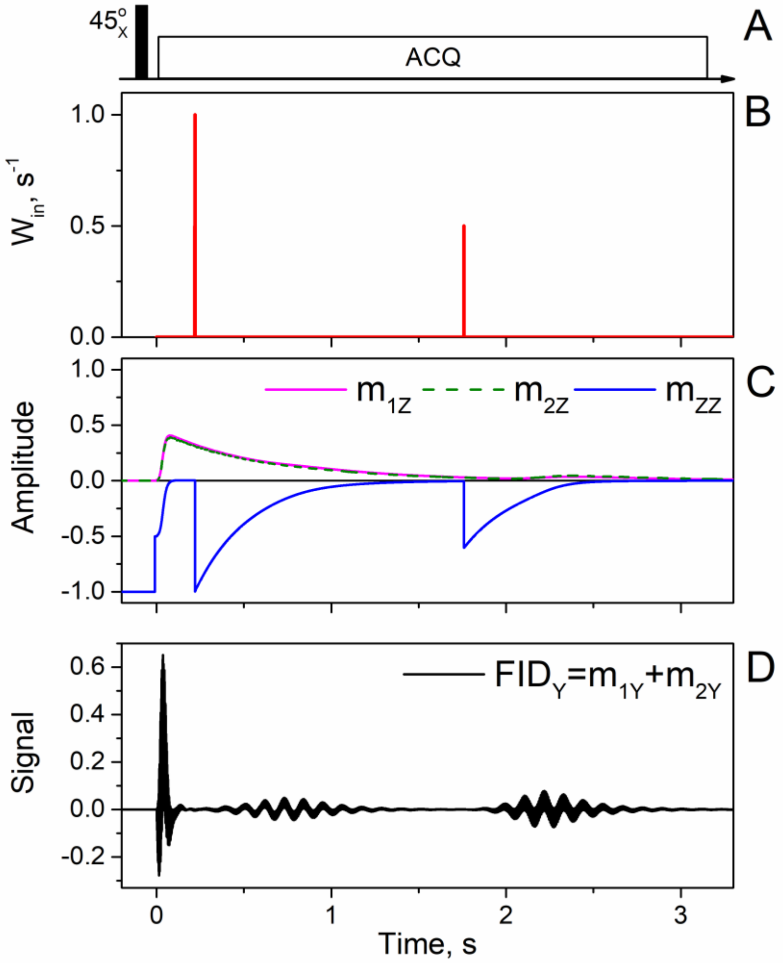

Figure S7. PASADENA simulations with spontaneous emission of "echo" like signals after sudden injection of polarization: sequence diagram (A), the hyperpolarization production rate, W_{in} represented with two delta functions (B), evolution of amplitudes of net m_{1Z} and m_{2Z} and multiplet m_{ZZ} magnetization as a function of time (C) and FID in corresponding PASADENA experiment (D). Radiation damping together with a multiplet polarization results in "spontaneous" emission of "echo" signals. Simulation parameters are: initial density matrix $\hat{\rho}(-0) = \hat{1}/4 - \hat{I}_{1Z}\hat{I}_{2Z}$, equilibrium state $\hat{\rho}_{eq} = \hat{1}/4$, J = 10 Hz, chemical shift difference 1.023 ppm, B_0=14.1 T; α_{RD} = 100 s^{-1}, relaxation times 0.6 s and operator of polarization source $\hat{\boldsymbol{S}} = W_{in}(t)[-\hat{I}_{1Z}\hat{I}_{2Z}]$. Depending on model parameters: relaxation rate, α_{RD}, $W_{in}(t)$ different number and shape of emitted signals can be obtained. Here we demonstrate that two instant additions of PASADENA polarization in the presence of weak transverse magnetization results in two "echo" like emitted signals.

10. Simulation: RASER emission as a result of an influx of negative net magnetization

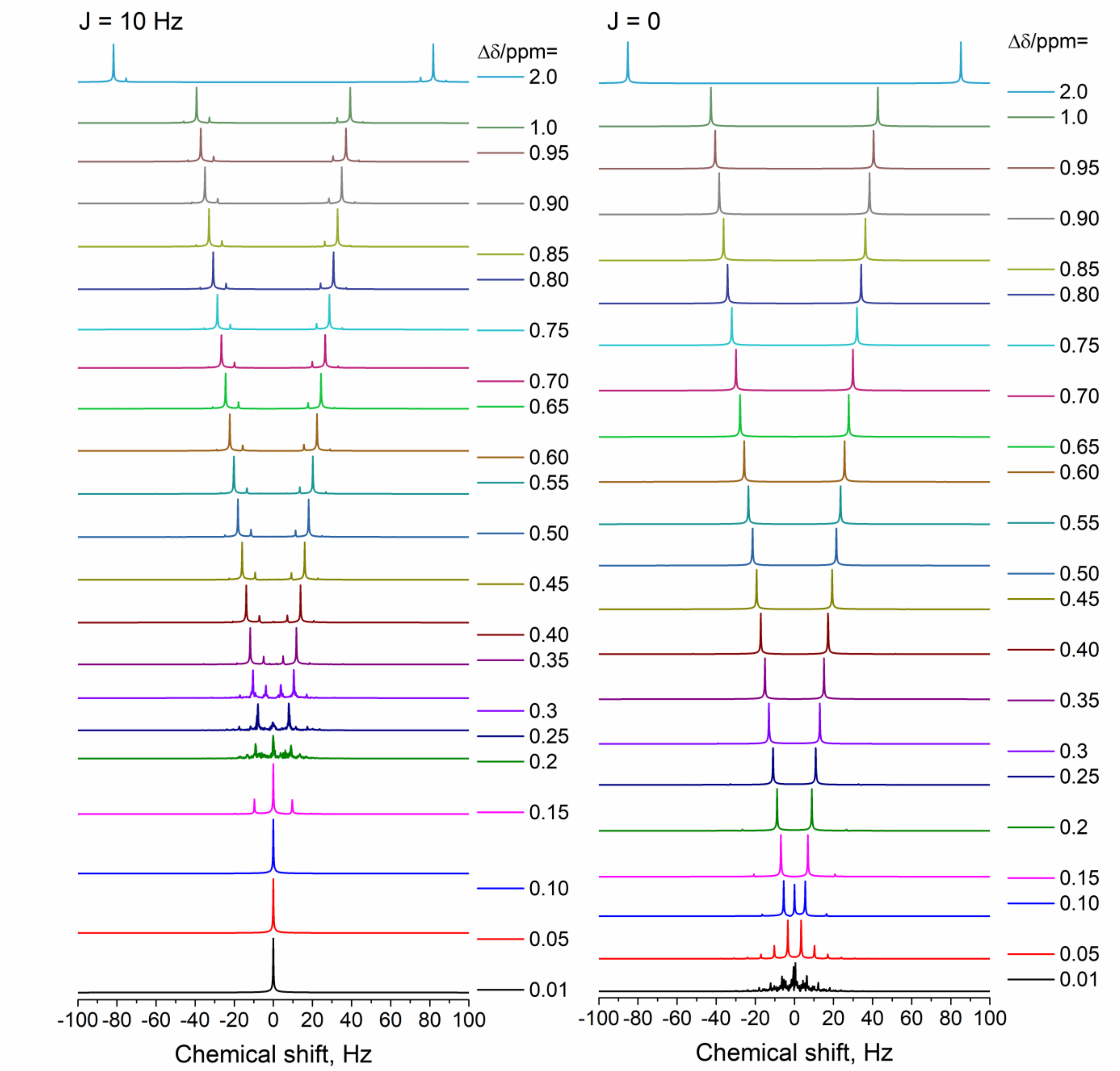

Figure S8. RASER spectra as a function of chemical shift difference. Simulation parameters: two spin-½ system, J = 10 Hz (left) and 0 (right), α_{RD} = 1000 s^{-1}, $\hat{S} = -0.25\left(\hat{I}_{1Z} + \hat{I}_{2Z}\right) s^{-1}$, relaxation times 5 s, B_0 = 1 Tesla. Coalescence of two RASER lines accompanies with an appearance of new lines. J-coupling constants are important for the description of the spin system in RASER conditions.

11. Simulation: PHIP-RASER spectra as a function of radiation damping amplitude

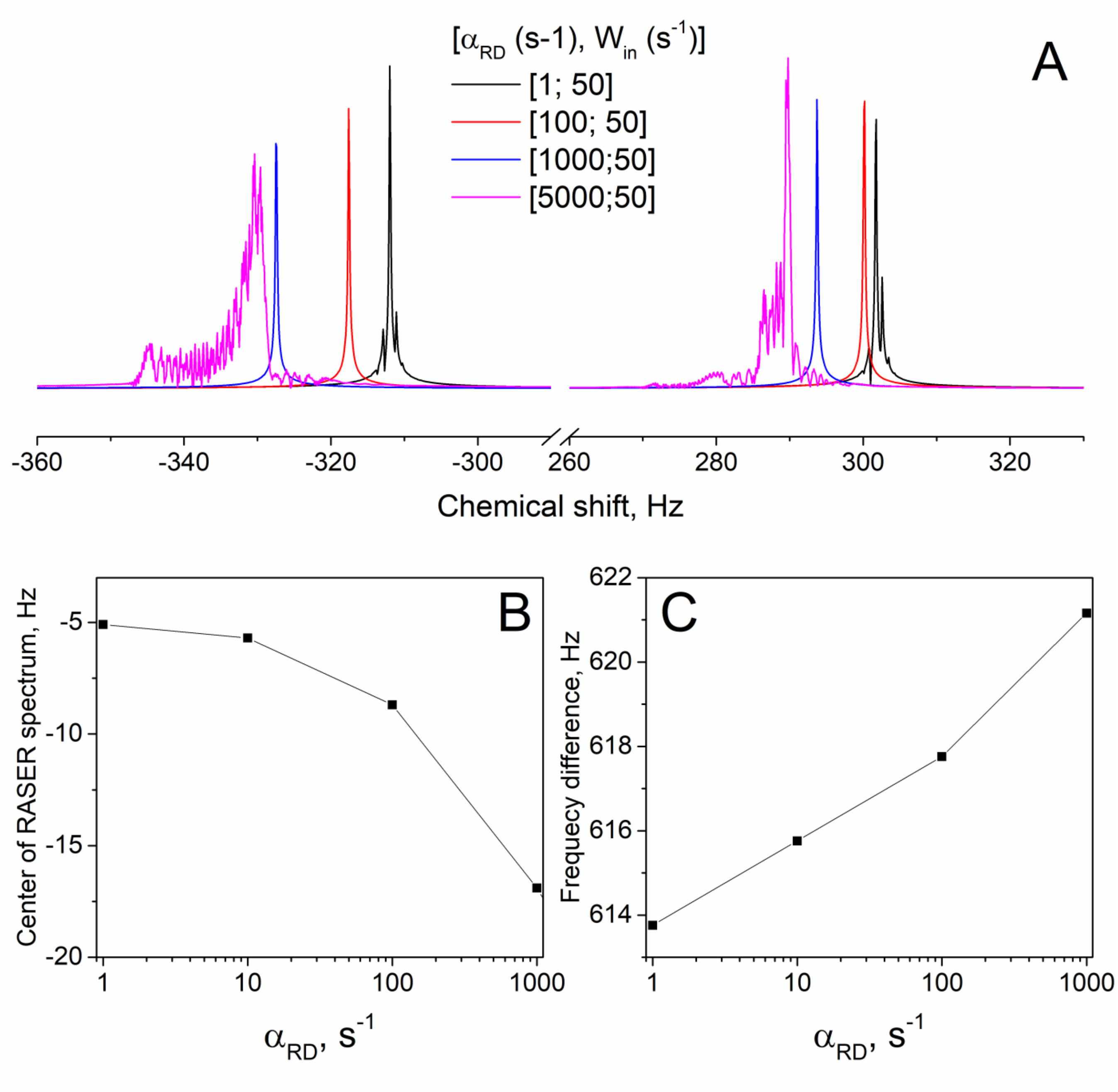

Figure S9. Effect of radiation damping amplitude on PHIP-RASER spectra. PHIP-RASER spectra (A), center frequency of PHIP-RASER spectrum and frequency difference of PHIP-RASER lines as a function of radiation damping, α_{RD}. Simulation parameters and calculation procedure the same as were used for Figure 3.

12. Experimental protocol 2: stop bubbling scheme

Protocol 2 (Scheme S1). *p*H_2 was supplied (bubbled) to the sample solution for τ_b = 5 s to hydrogenate EP (or MP) to generate PASADENA. Next, the bubbling was stopped and the system was allowed to settle down for a variable period of time, $\tau_w \geq 2$ s. After that, a rectangular 4 μs 45° RF-pulse was applied. Because *p*H_2 is immune to RF pulses, hydrogenation reaction of EP (or MP) with remaining dissolved *p*H_2 continues after RF-excitation. This experimental procedure allowed us to generate hyperpolarization after RF-pulse keeping high magnetic field homogeneity. All experiments were carried out at 25 °C and ambient pressure. During the experiments, some convection and diffusion occurred; the effect was neglected.

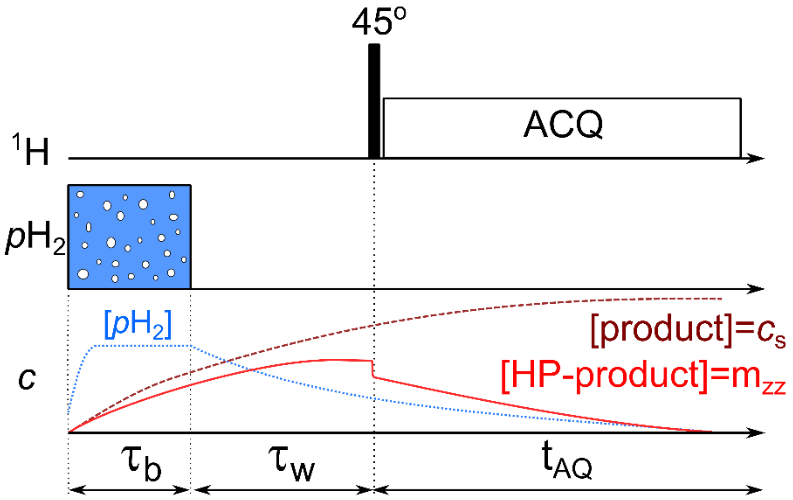

Scheme S1. Scheme of the experimental workflow (**protocol 2**): i) *p*H_2 supply for τ_b = 5 s with 0.2 bar overpressure. ii) waiting period 2 s < τ_w < 20 s; iii) excitation by hard 45° RF pulse and iv) signal acquisition (ACQ). Below, the concentrations of *p*H_2 (dotted blue line), total product ([product]=c_S, dashed wine line) and hyperpolarized product ([HP-product]=m_{zz}, solid red line) are plotted qualitatively. Note that hydrogenation with *p*H_2 continues after the RF excitation (*p*H_2 is immune to excitations with RF pulses).

13. Simulation: effect of radiation damping on PASADENA

These simulations (Fig. S10) show the difference of PASADENA dynamic between cases when radiation damping can be neglected and when it is much faster than T_2 relaxation rate.

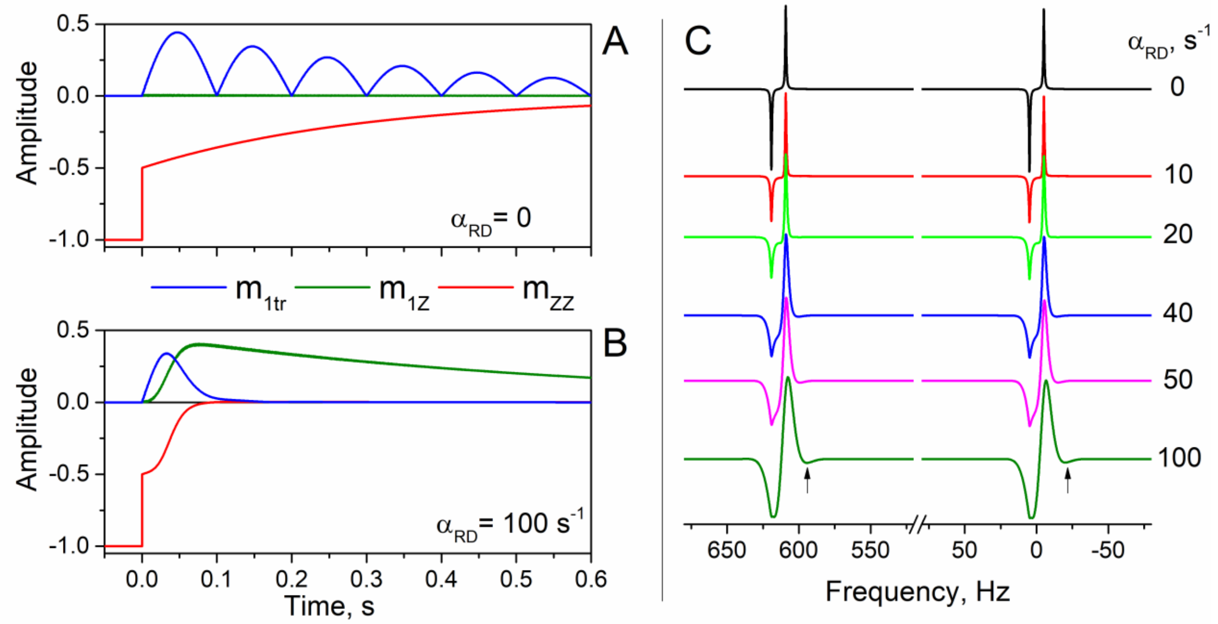

Figure S10. Simulations of PASADENA experiment with varying rate of radiation damping: evolution of transversal ($\mathrm{m}_{1tr} = \sqrt{\mathrm{m}_{1X}^2 + \mathrm{m}_{1Y}^2}$) and longitudinal ($m_{1Z}$) magnetizations and m_{ZZ} (amplitude of $\hat{\mathrm{I}}_{1Z}\hat{\mathrm{I}}_{2Z}$ spin order) for $\alpha_{RD} = 0$ (no RD, A) and $\alpha_{RD} = 100$ s^{-1} (B), and corresponding NMR spectra for α_{RD} = (0 – 100) s^{-1} (C). After 45° RF-excitation RD transforms remaining multiplet ZZ-polarization into net magnetization: $\hat{\mathrm{I}}_{1Z}\hat{\mathrm{I}}_{2Z} \xrightarrow{\mathrm{RD}} \hat{\mathrm{I}}_{1Z} + \hat{\mathrm{I}}_{2Z}$ (B), and also causes asymmetric line broadening (C). Appearance of extra deeps indicated by arrows. Initial density matrix is $\hat{\rho}(-0) = \hat{1}/4 - \hat{\mathrm{I}}_{1Z}\hat{\mathrm{I}}_{2Z}$. In equilibrium system is not polarized: $\hat{\rho}_{eq} = \hat{1}/4$. Simulation parameters are J = 10 Hz, chemical shift difference 1.023 ppm, B_0=14.1 T, W_{in}=0 and relaxation time 0.6 s.

14. Spontaneous PHIP-RASER bursts (protocol 2)

Interestingly, when RD is active, $\hat{I}_{1Z}\hat{I}_{2Z}$ spin order that remains after excitation, is quickly converted into longitudinal magnetization: $\hat{I}_{1Z}\hat{I}_{2Z} \xrightarrow{RD} \hat{I}_{1Z} + \hat{I}_{2Z}$ (Fig S11D [0, 0.1 s]). Transverse magnetization and resulting longitudinal magnetization relaxes with $\sim\tau_{RD}$ and ~T_1 times respectively. The fast decay of transverse magnetization reveals itself in a broadening of the spectral lines, which is in fact asymmetric: emission lines are broader and less intense than absorption lines (Fig S11C,E). When this asymmetry is observed in the experiment, it is a first indication of the presence of RD in PASADENA experiments. If undesired, special care should be taken, e.g. detune probe (reduce Q), reduce field homogeneity or use pulse sequences with dephasing gradients.[13,14]

Another, even more important aspect occurs later. If *p*H_2 is still present after 45° RF-excitation, then hydrogenation reaction and hence production of PASADENA polarization continuous.

We found “spontaneous” bursts of RASER with an echo-like appearance up to 30 s after finishing *p*H_2 bubbling and consequent excitation with 45° RF-pulse (Fig S11B). The echo like signals manifest itself as a very narrow “artifacts” on an emission spectral lines (compare Fig. S11C-8 and 9).

The experiment was repeated >20 times without changing the sample, and the observations were reproduced. However, during the experiment the concentration of substrate and the activity of catalyst are changing. This results in differences in the number of appearing echo like signals and their positions.

The observations were qualitatively reproduced by simulating a coupled spin system with RD and source of hyperpolarization (Fig S11D,E). Transverse magnetization via RD excites multiplet spin order of PASADENA ($\hat{I}_{1Z}\hat{I}_{2Z}$) and converts it into longitudinal and transverse magnetization. The latter manifests itself as a “spontaneous” emission of echo like signal. The amplitude of emitted signals decay from burst to burst because *p*H_2 is consumed and hence polarization production rate decays.

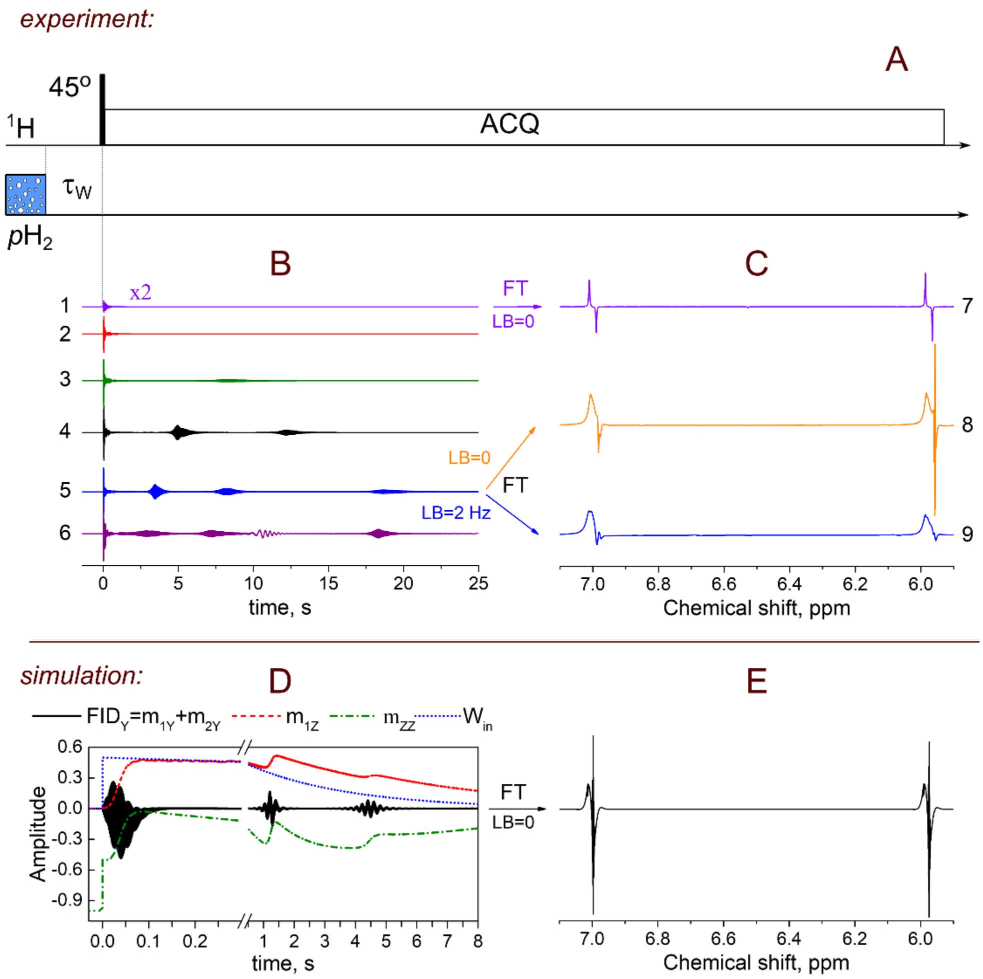

Figure S11. PASADENA experiment with spontaneous emission of "echo" like signals: sequence diagram (A), experimental observations (B), simulated evolution of PASADENA (m_k is polarization level of spin order k=$\hat{I}_{1Y}$, $\hat{I}_{2Y}$, $\hat{I}_{1Z}$ and $\hat{I}_{1Z}\hat{I}_{2Z}$) (D) and corresponding NMR spectra (C and E). Six ^{1}H NMR free induction decays (FIDs) were acquired after 45° excitation of the sample that was presaturated with pH_2 each time before RF-excitation. Concentration of substrate and catalyst activity decreases from (6) to (2), $\tau_w = 1$ s. FID (1) was multiplied by a factor of 2 for a better visual perception, $\tau_w = 25$ s. Spectra (8-9) are Fourier transformation (FT) of FID (5) with line broadening (LB) 0 and 2 Hz respectively. Note the "spontaneous" emission of PASADENA and RD is more profound on emission spectral components; absorption components are predictably broadened. Simulation parameters are: initial density matrix $\hat{\rho}(-0) = \hat{1}/4 - \hat{I}_{1Z}\hat{I}_{2Z}$, equilibrium state $\hat{\rho}_{eq} = \hat{1}/4$, J = 10 Hz, chemical shift difference 1.023 ppm, B_0=14.1 T, α_{RD} = 100 s^{-1}, relaxation time 5 s and rate of polarization influx $W_{in} = 0.5e^{0.3s^{-1}\cdot t}$. Depending on model parameters: relaxation rate, α_{RD}, $W_{in}(t)$ different number and shape of emitted signals can be obtained.

References

[1] A. Abragam, *The Principles of Nuclear Magnetism*, Clarendon, **1961**.
[2] N. Bloembergen, R. V. Pound, *Phys. Rev.* **1954**, *95*, 8–12.
[3] O. W. Sørensen, G. W. Eich, M. H. Levitt, G. Bodenhausen, R. R. Ernst, *Prog. Nucl. Magn. Reson. Spectrosc.* **1984**, *16*, 163–192.
[4] R. Freeman, S. Wittekoek, R. R. Ernst, *J. Chem. Phys.* **1970**, *52*, 1529–1544.
[5] K. Ivanov, A. Yurkovskaya, H.-M. Vieth, *J. Chem. Phys.* **2008**, *129*, 234513.
[6] A. N. Pravdivtsev, A. V. Yurkovskaya, H.-M. Vieth, K. L. Ivanov, *J. Chem. Phys.* **2014**, *141*, 155101.
[7] S. Appelt, A. Kentner, S. Lehmkuhl, B. Blümich, *Progress in Nuclear Magnetic Resonance Spectroscopy* **2019**, DOI 10.1016/j.pnmrs.2019.05.003.
[8] M. Suefke, S. Lehmkuhl, A. Liebisch, B. Blümich, S. Appelt, *Nat. Phys.* **2017**, *13*, 568–572.
[9] A. N. Pravdivtsev, J.-B. Hövener, *Chemistry – A European Journal* **n.d.**, *0*, DOI 10.1002/chem.201806133.
[10] H.-Y. Chen, Y. Lee, S. Bowen, C. Hilty, *J. Magn. Reson.* **2011**, *208*, 204–209.
[11] A. N. Pravdivtsev, J.-B. Hövener, *MOIN Spin Library*, (Www.Moincc.De/Method-Development/Mr/Moin-Spin-Library) UKSH, University Kiel, **2018**.
[12] X.-A. Mao, C.-H. Ye, *Concepts Magn. Reson.* **1997**, *9*, 173–187.
[13] J. A. Aguilar, R. W. Adams, S. B. Duckett, G. G. R. Green, R. Kandiah, *Journal of Magnetic Resonance* **2011**, *208*, 49–57.
[14] A. N. Pravdivtsev, F. Sönnichsen, J.-B. Hövener, *J. Magn. Reson.* **2018**, *297*, 86–95.
